# Antibiotic prophylaxis in patients who had undergone to prostate biopsy in between the EMA warning era: effects of fluoroquinolones in diabetic and non-diabetic patients. Results of an observational cohort study

**DOI:** 10.1007/s00345-022-04055-7

**Published:** 2022-06-10

**Authors:** Riccardo Bartoletti, Francesco Claps, Gabriele Tulone, Alessandro Perotti, Alessandro Zucchi, Niccolò Riccardi, Vincenzo Ficarra, Cosimo De Nunzio, Andrea Tubaro, Alchiede Simonato

**Affiliations:** 1 Department of Translational Research a New Technologies in Medicine and Surgery, University of Pisa, Pisa, Italy; 2 Department of Medical, Surgical and Health Sciences, University of Trieste, Trieste, Italy; 3 Department of Surgical, Oncological and Stomatological Sciences, University of Palermo, Palermo, Italy; 4 Infectivology Unit, Azienda Ospedaliero Universitaria Pisana, Pisa, Italy; 5 Department of Surgery, University of Messina, Messina, Italy; 6 Department. of Urology, University Rome La Sapienza, Rome, Italy; 7 Urology Unit, Cisanello Hospital, Azienda Ospedaliero Universitaria Pisana, Bld 30, F Orange Route, room 275, Via Paradisa 2, 56124 Pisa, Italy

**Keywords:** Prostate biopsy, Infection, Antibiotic prophylaxis, Fluoroquinolones, Antibiotic resistance

## Abstract

**Purpose:**

To investigate the effects of different antibiotic prophylaxis regimens in patients with diabetes mellitus (DM) candidates to trans-rectal ultrasound-guided prostate biopsy (TRUSPB).

**Methods:**

143 outpatients with DM who underwent TRUSPB during the period 2018–2020 were selected from a cohort of 1150 patients in 3 different institutions. Exclusion criteria were allergies, concomitant anti-platelet therapies and uncontrolled DM. Different antibiotic prophylaxis regimens were adopted. Bacterial resistance levels to fluoroquinolones into the different communities were also collected. Univariable and multivariable binomial logistic regression analyses were used to assess the odds ratio (OR) with 95% confidence intervals (CIs) testing the risk of infective complications' occurrence after adjusting for clinical covariates.

**Results:**

Overall, DM patients were significantly associated with infective complications' occurrence (*p* < 0.001). No differences on the event of sepsis were found between diabetic and non-diabetic patients. Clinically relevant infections with fever > 37 °C were found in 9.1% and 1.5% (*p* < 0.001) in diabetic and non-diabetic patients, respectively. Trimethoprim–sulphametoxazole and fluoroquinolones were six times more efficient than Cefixime in non-diabetic patients. Fluoroquinolones confirmed the same effect in diabetic patients although the level of resistance in the period of study decreased only from 56 to 46%.

**Conclusion:**

Fluoroquinolones were active in antibiotic prophylaxis of diabetic patients who had undergone to TRUSPB independently from the level of bacterial resistance found in the community. These results conflict with the recent European warning and support the Japanese and American guidelines on the topic.

**Supplementary Information:**

The online version contains supplementary material available at 10.1007/s00345-022-04055-7.

## Introduction

Urinary tract infections (UTIs) pose a serious threat to patients with diabetes mellitus (DM). Different authors demonstrated that the number of hospitalizations for pyelonephritis treatment and the recurrence of cystitis are more frequent in patients with DM [1]. In addition, recent studies highlighted that prolonged use of a type of hypoglycemic drugs, such as sodium–glucose co-transporter-2 (SGLT2) inhibitors, may increase the prevalence of UTIs [2]. Trans-rectal ultrasound-guided prostate biopsy (TRUSPB) has become the most confident method for the urologist to obtain a definite response regarding early diagnosis of prostate cancer [3]. Complications after TRUSPB, such as hematuria, UTIs, acute urinary retention, lower urinary tract symptoms, have been previously described. In detail, the incidence of UTIs in patients who received TRUSPB is considerably higher than in patients who received the alternative trans-perineal approach, even if urologists are generally less confident with this technique [4, 5]. TRUSPB-related infections may lead to prolonged hospitalization as well as increased morbidity and mortality. Increased rates of infective complications after TRUSPB requiring adjunctive antibiotic treatment from 6.1 to 9.7% were found during the period 2010–2019 in Europe. In detail, 2.8% of patients developed febrile infections and 4% of them required hospitalization, while 0.12% died after TRUSPB due to sepsis [6].

Wu et al. found that body mass index, DM and preoperative characterization were independent risk factors for infection in patients who underwent TRUSPB [7]. Ding et al. recently reported the results of a univariate and multivariate logistic regression analysis on 2192 patients who underwent trans-perineal prostate biopsy. Patients with DM and history of urinary retention were more likely to have infective complications. In detail, about 34% of investigated patients had DM and infective complications were found in 1.87% of cases. Multivariate analysis highlighted an increased objective risk of complications related to DM of 2.037 times [3].

Current recommendations for antibiotic prophylaxis in candidates to TRUSPB vary in relation to national and international guidelines. The European Association of Urology (EAU) guidelines, recommended the use of povidone-iodine disinfection and targeted or augmented prophylaxis since the European Medicine Agency (EMA) warning forbad the use of Fluoroquinolones, while both the Japanese Urology Association (JUA) and American Urology Association (AUA) guidelines (last versions 2015 and 2017 respectively), still supported the use of fluoroquinolones as first choice antibiotic prophylaxis [8–11].

Hence, we retrospectively analyzed the clinical data of a multi-center cohort study subgroup of 143 diabetic patients selected from a series of 1150 patients who underwent TRUSPB between 2017 and 2019 [12] to investigate the effects of different types of antibiotic prophylaxis on symptoms suggestive of UTIs through the period of the EMA warning about the use of fluoroquinolones for the antibiotic prophylaxis of patients candidates to TRUSPB [13]. Data related to the level of antibiotic resistance to Fluoroquinolones along the period of the study have also been analyzed in each single center involved in the project.

## Materials and methods

The study design consists of a retrospective multi-institutional cohort of patients who received TRUSPB during the period 2018–2020. One hundred forty three patients with DM were selected from a cohort of 1150 patients for a subgroup analysis.

Adjunctive information was collected regarding the concomitant medical therapies, co-morbidities and allergies. Patients with reported allergies to specific type of food, beverages and pharmacological compounds were excluded from the study. Similarly, patients with secondary prevention therapies for thromboembolism were excluded from the study. Abdominal ultrasound was performed independently from trans-rectal ultrasound investigations and urine culture was also collected before the biopsy to exclude the risk of concomitant urinary tract infections.

Different antibiotic prophylaxis regimens were adopted according to the protocols released from the local ethical committee of each single participating center. The antibiotic prophylaxis treatment regimen included the administration of each dose the night before the biopsy, 2 h to half an hour before the biopsy and 24 and 48 h after the biopsy according to the EAU guidelines [8, 9].

Different antibiotic prophylaxis regimens have been adopted and in particular Cefixime, trimethoprim–sulphametoxazole, levofloxacin, prulifloxacin, ciprofloxacin and the augmented prophylaxis with ceftriaxone/fosfomycin. Dosage of antibiotic compounds was adapted to creatinine clearance and estimated glomerular filtration rate (eGFR) for each single patient. Information regarding short- and long-term complications after the biopsy, unplanned visits or hospital readmission due to different symptoms or clinical signs were also collected. Adjunctive data on the different rates of antibiotic resistance to antibiotics and fluroquinolones in particular into the different communities of patients selected for the study along the period 2017–2019 have been also registered. The aim was to compare different types of antibiotic prophylaxis administered to the increased risk of infective complications after TRUSPB. All patients enrolled received from 12 to 16 core biopsies. General status, age, co-morbidities and co-medications were considered as relevant factors for the number of prostate biopsy cores taken from each single patient enrolled in the study.

Descriptive analysis included frequencies and proportions for categorical variables. Medians and interquartile range (IQR) were reported for continuous coded variables. Mann–Whitney U or Kruskal–Wallis test was used for comparison of the continuous data and the Chi-square or Fisher’s exact test for categorical data. All tests were two-sided with a level of significance set at *p* < 0.05. Univariable and multivariable binomial logistic regression models were used to assess the odds ratio (OR) with 95% confidence intervals (CI) testing the risk of infective complications after adjusting for each pre-biopsy covariates. These included age at biopsy, year of biopsy, number of cores retrieved, antibiotic prophylaxis regimen adopted and presence of DM. After univariable analysis, factors with *p* < 0.2 were entered into the multivariable model, followed by backward elimination to determine the factors most associated with the infective complications' occurrence. The current study represents a sensitivity analysis on a subgroup of patients with DM extracted from a large multi-center collaboration. Susceptibility tests were obtained from urine of patients who developed infective complications. Eucast breakpoints range were also determined. More efficient antibiotics were vancomicin, aminoglycosides, cephalosporins and fluoroquinolones. Cases of complicated UTIs or sepsis were handled by the Infectious Diseases Department at each participating center. Statistical analyses were performed using RStudio v.1.2.5001 (Integrated Development for RStudio). The study reports have been collected according to the STROBE statement.

## Results

### Patients and clinical characteristics

Descriptive baseline characteristics and clinico-pathological features for the general cohort of 1150 patients stratified by the presence of DM are represented in Table 1. Median age of patients at TRUSPB was 70 (IQR, 64–76) years with a median pre-biopsy prostate-specific antigen (PSA) of 7.4 ng/ml. Median number of biopsy cores taken was 12 (IQR, 12–16). The most relevant complications were hemospermia and hematuria (11% of cases), rectal bleeding (7.5%), rectal pain (6.3%), fever > 37 °C (2.4%), urinary retention (1.5%) sepsis (0.8%). DM was a surrogate endpoint of the study and due to these reasons considered for a subgroup analysis. About 41.5% of patients received antibiotic prophylaxis with fluoroquinolones, 23.1% trimethoprim–sulfamethoxazole, 20.7% augmented prophylaxis with ceftriaxone and fosfomycin, 14.6% Cefixime. Unplanned visits and hospital readmission after the biopsy were 2.5 and 0.9% respectively.

**Table 1:** Descriptive baseline characteristics and clinico-pathological features for the cohort of 1150 patients who underwent trans-rectal ultrasound prostate biopsy according to DM status

Variable	Overall cohort	Diabetes		*P*
		No	Yes	
Patients, *n*. (%)	1150 (100.0)	1007 (87.6)	143 (12.4)	
Age (years), median (IQR)	70 (64–76)	70 (64–75)	70 (65–76)	0.25
No. of cores, median (IQR)	12 (12–16)	12 (12–16)	12 (12–16)	0.86
Year of prostate biopsy, *n*. (%)				0.25
2018	227 (19.7)	203 (20.2)	24 (16.8)	
2019	726 (63.1)	638 (63.4)	88 (61.5)	
2020	197 (17.1)	166 (16.5)	31 (21.7)	
Antibiotic prophylaxis’ regimen, *n*. (%)				0.03
Cefixime 400 mg	168 (14.6)	153 (15.2)	15 (10.5)	
Ceftriaxone 1 g—Fosfomycin 3 g	238 (20.7)	207 (20.6)	31 (21.7)	
Trimethoprim 160 mg—Sulfamethoxazole 800 m	266 (23.1)	239 (23.7)	27 (18.8)	
Ciprofloxacin 500 mg	25 (2.2)	17 (1.7)	8 (5.6)	
Levofloxacin 500 mg	443 (38.5)	382 (37.9)	61 (42.7)	
Prulifloxacin 600 mg	10 (0.9)	9 (0.9)	1 (0.7)	
Infective complications, *n*. (%)	30 (2.6)	17 (1.7)	13 (9.1)	< 0.001
Specific complications, *n*. (%)				
Haematospermia	126 (11.0)	107 (10.6)	19 (13.3)	0.42
Haematuria	126 (11.0)	105 (10.4)	21 (14.7)	0.17
Rectal bleeding	86 (7.5)	74 (7.4)	12 (8.4)	0.78
Fever (> 37 °C)	28 (2.4)	15 (1.5)	13 (9.1)	< 0.001
Sepsis	9 (0.8)	6 (0.6)	3 (2.1)	0.16
Urinary retention	17 (1.5)	14 (1.4)	3 (2.1)	0.78
Rectal pain	73 (6.3)	55 (5.5)	18 (12.6)	0.002
Unplanned visit, *n*. (%)	29 (2.5)	25 (2.5)	4 (2.8)	0.9
Unplanned readmission*, n*. (%)	10 (0.9)	7 (0.7)	3 (2.1)	0.23
Center, *n.* (%)				< 0.001
Palermo (Center A)	130 (11.3)	96 (9.5)	34 (23.8)	
Cuneo (Center B)	621 (54.0)	557 (55.3)	64 (44.8)	
Pisa (Center C)	399 (34.7)	354 (35.2)	45 (31.5)	

*QR* interquartile range

Diabetic patients were 143 (12.4%) and DM was pharmacologically controlled at biopsy. All of them had negative urine culture and none previous antibiotic therapies within the last 3 weeks. DM patients showed a higher risk of developing clinically relevant UTIs with fever > 37 °C (9.1% vs 1.5%; *p* < 0.001) and rectal pain (12.6 vs 5.5%; *p* 0.002) after the TRUSPB. No significant differences on the event of sepsis have been found between the two groups. Forty-nine percent of diabetic patients and 41.5% of non-diabetic patients received fluoroquionolones as antibiotic prophylaxis respectively (*p* = 0.03). The prevalence of infective complications and rectal pain after the biopsy was significantly higher in diabetic than non-diabetic patients.

### Risk factors associated with infective complications after TRUSPB

Overall, 30 (2.6%) infective complications were found: 17 (1.7%) and 13 (9.1%) among non-DM and DM patients (*p* < 0.001). Univariable and multivariable binomial logistic regression analyses adjusted for the effect of clinical covariates among non-diabetic and diabetic patients are presented in Table 2. Univariable analysis confirmed that the number of biopsy cores has to be considered as a relevant factor for developing infective complications after TRUSPB in both cohorts. A 1.29- and 1.77-fold increased risk in non-diabetic and diabetic patients was described (*p* = 0.041 and *p* = 0.004 respectively).

**Table 2:** Univariable and multivariable binomial logistic regression analysis for prediction of infective complications among 1007 non-diabetic and 143 diabetic patients who underwent TRUSPB

Variable	Univariable analysis		Multivariable analysis	
	OR (95% CI)	*p*	OR (95% CI)	*p*
Non-diabetic cohort				
Age (years), as cont	0.99 (0.93–1.05)	0.7	–	–
No. of cores, as cont	1.29 (1.04–1.69)	0.041	1.12 (0.88–1.64)	0.5
Year of prostate biopsy				
2018	1.00 (Ref.)	–	–	–
2019	2.25 (0.62–14.4)	0.3		
2020	0.61 (0.03–6.41)	0.7		
Antibiotic prophylaxis’ regimen				
Cefixime	1.00 (Ref.)	–	1.00 (Ref.)	–
Ceftriaxone–Fosfomycin	0.61 (0.17–2.05)	0.4	0.62 (0.18–2.09)	0.4
Trimethoprim–Sulfamethoxazole	0.10 (0.01–0.61)	0.036	0.16 (0.01–1.25)	0.12
Fluoroquinolones	0.30 (0.09–1.02)	0.052	0.46 (0.10–2.38)	0.3
Diabetic cohort				
Age (years), as cont	0.95 (0.88–1.02)	0.34	–	–
No. of cores, as cont	1.77 (1.25–2.79)	0.004	1.17 (0.93–1.62)	0.3
Year of prostate biopsy				
2018	1.00 (Ref.)	–	–	–
2019	3.29 (0.59–61.6)	0.3		
2020	0.77 (0.03–20.1)	0.9		
Antibiotic prophylaxis’ regimen				
Cefixime	1.00 (Ref.)	–	1.00 (Ref.)	
Ceftriaxone–Fosfomycin	0.22 (0.05–0.95)	0.04	0.21 (0.04–0.92)	0.04
Trimethoprim–Sulfamethoxazole	0.12 (0.02–0.63)	0.01	0.43 (0.03–5.68)	0.5
Fluoroquinolones	0.02 (0.00–0.15)	< 0.001	0.07 (0.00–0.70)	0.048

*TRUSPB* transrectal prostate biopsy, *OR* odds ratio, *CI* confidence interval

The univariable analysis of different compounds adopted for antibiotic prophylaxis regimens evidenced a significant effect of trimethoprim–sulfametoxazole (*p* = 0.003) and fluoroquinolones (*p* < 0.001) among overall cohort (about six times more protective if compared to Cefixime) while this effect seems to be significantly reduced in favor of fluoroquinolones in DM patients. Augmented antibiotic prophylaxis (Ceftriaxone + Fosfomycin) seems to be more efficient in diabetic than non-diabetic patients.

Multivariable analysis confirmed these findings on non-diabetic patients while fluoroquinolones maintain their effects on patients with DM (about 5 times more efficient than Cefixime) but not trimethoprim–sulphametoxazole.

Although the bacterial resistance to fluoroquinolones decreased along the period of the study into the communities located in the north, center and south areas of the country respectively, these variations are not statistically significant and did not influence the response of patients to antibiotic prophylaxis (Figure S1). No adverse events have been registered among the enrolled patients related to the type, dosage and timing of administration of each single antibiotic compound.

## Discussion

### Infectious complications and antibiotic prophylaxis

The rate of infectious complications after TRUSPB varies from 1.9 to 27.7% according to different countries and showed a trend to increase in the last few years [14, 15]. The Global Prevalence Study of Infections in Urology (GPIU) confirmed that antibiotic prophylaxis often showed scarce efficacy due to antibiotic resistant bacteria [6]. Nevertheless, the comparative analysis of two different periods (2010–2014 and 2016–2019) demonstrated that most part of patients reported bothersome lower urinary tract symptoms, such as frequency and urgency, but not fever or sepsis. In particular, the rate of symptomatic cases with positive urine culture after TRUSPB raised from 1.5 to 2.4% in the two periods as well as the number of *E. coli* isolates. No statistically significant differences in terms of lethal cases and patients who required hospitalization or intensive care ward after TRUSPB were found. The bacterial resistance to antibiotics raised from 4.2 to 12.7% in the two periods, but those to fluoroquinolones, in particular, decreased from 1.3 to 0.2% probably related to the EMA warning, suggesting the limited use of these compounds in the clinical practice to avoid significant adverse events [13]. Anyhow fluoroquinolones were adopted as preferred antibiotic therapy in the case of symptoms after TRUSPB in the two periods. Similarly, the level of bacterial resistance to fluoroquinolones in the 3 centers involved in our study varied during the study period with a slight increase from 2017 to 2018 and a significant decrease in 2019, thus confirming the effects of the EMA warning. Paradoxically, univariable analysis of different antibiotics adopted for prophylaxis of TRUSPB confirmed the effects of Fluoroquinolones as six times more protective against infections than Cefixime, and without significant adverse events. The same results have been obtained in multivariable analysis on patients with DM.

Other antibiotic treatments such as the augmented prophylaxis with the combination of ceftriaxone and fosfomicin and trimethoprim–sulphametoxazole showed good results in terms of efficacy, but supposedly less efficient than fluoroquinolones. Carignan et al. in the context of a comparative study on 9391 patients who underwent to TRUSPB and received different antibiotic prophylaxis with ciprofloxacin or fosfomicin found that fosfomicin is not an effective alternative to ciprofloxacin to prevent urinary sepsis [16]. On the other hand, there is not a rational explanation regarding the superior efficacy of trimethoprim–sulphametoxazole in non-diabetic other than in diabetic patients. Fromtling et al. experimentally described these results in normal and diabetic mice [17].

Considering the non-diabetic cohort, no differences were found about the antibiotic prophylaxis’ regimen adopted at multivariable analysis. This could be explained by the low rates of infective complications (1.7%) in this subgroup of patients. However, these findings might represent a surrogate of no need of augmented prophylaxis in such cohort of TRUSPB candidates. Conversely, DM patients were significantly more exposed to infectious complications after TRUSPB compared to their counterpart. Our experience confirmed these results. The increased risk is also related to the number of biopsy core received. In vitro studies on *E. coli* strains demonstrated that bacteria grow faster in urine with higher concentrations of glucose although no well-designed clinical studies confirmed this hypothesis [18]. Hyperglycemia impaired cytokine production and immune response from the host and increased adherence of type-1 fimbriated *E.coli* strains to uro-epithelial cells in women with DM [19–21].

### Bothersome symptoms after TRUSPB

Other significant bothersome symptoms after the biopsy were represented by rectal pain and urinary retention. No significant relationship between the type of antibiotic prophylaxis administered and short-term adverse events was found.

Within a retrospective multi-center observational study including 3350 patients undergoing TRUSPB following three different management protocols, Perán Teruel et al. found that adoption of augmented antibiotic prophylaxis was the most significant variable for no complications and rectal pain occurrence. Furthermore, at multivariable analysis, the authors confirmed the independent effect of age, antibiotic prophylaxis and pre-TRUSPB PSA on the occurrence of infections [22]. Rectal pain after TRUSPB resulted more frequent in diabetic than non-diabetic patients (*p* = 0.002). TRUSPB may generate significant local pain and discomfort associated with both the insertion and movements of ultrasound probe in the rectum and the core-biopsy needle puncture [23]. Since the number of prostate biopsy cores performed of our series of patients was comparable in the 3 referral centers, rectal pain seemed mainly due to the mechanical stimulation of the rectal wall. The rectum is innervated by splanchnic sensory and somatic nerves up and down the dentate line, respectively. Somatic innervations fibers derived from the inferior rectal nerve and represent an extremely pain-sensitive area in correspondence of the prostate apex [24]. Rectal pain was found more frequent in diabetic patients compared their counterpart. The difference may be justified by the presence of neuropathic pain that may occur as forms of hyperalgesia (amplified pain from mild nocuous stimuli) or allodynia (pain caused by innocuous stimuli) [25].

Bladder outlet obstruction (BOO) in patients who underwent TRUSPB is related to different factors, such as the entity of obstructive urinary symptoms and the previous use of alpha blockers. Mechanical stimulation of the prostate by TRUSPB may generate local edema and inflammation but not partial or complete acute urinary retention episodes. Sefik et al. analyzed the results obtained in a randomized controlled trial on 112 candidates to TRUSPB. The study group patients received Tamsulosin 1 week before the biopsy and continued the therapy 1 week after. Patients of the control group did not receive adjunctive medical therapies. They found significant differences in terms of International Prostate Symptom Score (IPSS), maximum flow rate at uroflowmetry and post-void residual urine volume in favor of patients included in the study group [26].

The retrospective fashion of the study design represents a significant limitation of the study, although all findings regarding patients with DM perfectly fitted with those found in the international literature.

## Conclusion

The results obtained are attractive due to the possible persistent active role of Fluoroquinolones in the antibiotic prophylaxis of patients’ candidates to TRUSPB as indicated by the JUA and the AUA. Fluoroquinolones were more active than other antibiotics monotherapies or augmented therapies mainly in diabetic subjects to decrease the incidence of UTIs after procedure, independently from antibiotic resistance levels found in the communities during the period of the study across the EMA warning regarding the restricted use of these compounds to avoid the risk of adverse events. However, local epidemiology of bacterial resistance to Fluoroquinolones and previous history of patient’s exposure to Fluoroquinolones therapy should be always assessed for planning an effective antibiotic prophylaxis in patients’ candidates to TRUSPB.

## Supplementary Information

Below is the link to the electronic supplementary material.

Supplementary file1 (DOCX 22 KB)

## Data Availability

Not applicable.

## Material availability

Materials included in the present work have not been previously published in other journals.
