# Supplementary material for: Antibiotic prophylaxis in patients who had undergone to prostate biopsy in between the EMA warning era: effects of fluoroquinolones in diabetic and non-diabetic patients. Results of an observational cohort study
Source: World J Urol. 2022 Jun 10;40(8):2025–31. doi: 10.1007/s00345-022-04055-7 (PMC9279202; doi:10.1007/s00345-022-04055-7)
Supplement: Supplementary file 1 — Supplementary file1 (DOCX 22 KB) [file 345_2022_4055_MOESM1_ESM.docx]

**Supplementary Figure 1**. Distribution of antibiotic resistance to Fluoroquinolones profile in the communities of 3 referral centers during the period of the study. Resistance level decreased slowly from 2018.
